# Resource Allocation for Edge Computing without Using Cloud Center in Smart Home Environment: A Pricing Approach

**DOI:** 10.3390/s20226545

**Published:** 2020-11-16

**Authors:** Huan Liu, Shiyong Li, Wei Sun

**Affiliations:** School of Economics and Management, Yanshan University, Qinhuangdao 066004, China; liuhuan5011@foxmail.com (H.L.); wsun@ysu.edu.cn (W.S.)

**Keywords:** edge computing, smart homes, resource pricing, resource allocation, utility optimization

## Abstract

Recently, more and more smart homes have become one of important parts of home infrastructure. However, most of the smart home applications are not interconnected and remain isolated. They use the cloud center as the control platform, which increases the risk of link congestion and data security. Thus, in the future, smart homes based on edge computing without using cloud center become an important research area. In this paper, we assume that all applications in a smart home environment are composed of edge nodes and users. In order to maximize the utility of users, we assume that all users and edge nodes are placed in a market and formulate a pricing resource allocation model with utility maximization. We apply the Lagrangian method to analyze the model, so an edge node (provider in the market) allocates its resources to a user (customer in the market) based on the prices of resources and the utility related to the preference of users. To obtain the optimal resource allocation, we propose a pricing-based resource allocation algorithm by using low-pass filtering scheme and conform that the proposed algorithm can achieve an optimum within reasonable convergence times through some numerical examples.

## 1. Introduction

With the emergence and rapid development of the Internet of Things (IoT), Internet technology is moving towards the direction of “intelligence of everything” on the basis of “Internet of everything (IOE)” [1]. People’s quality of life is steadily improving and they are also looking for more convenient and comfortable services due to technological progress. Currently, smart home applications represented by smart speakers, sweeping robots and smart air conditioners are becoming an indispensable part in many users’ lives [2]. In China, the scale of smart home market is growing at a rate of 20% to 30% per year. According to Prospective Industrial Research Institute, China’s smart home market is expected to reach ¥436.9 million in 2021 [3]. A mushrooming number of smart home applications are entering into millions of homes, from smart washing machines to smart vacuum cleaners, from smart door locks to telemedicine, all of them reflect the charm and potential of technology. The new technology of smart homes and Internet of Things would meet people’s pursuit of “livable, comfortable, convenient and safe” living environment [4].

The Smart Grid (SG) is considered as an imminent future power network due to its fault identification and self-healing capabilities. As one of the most significant terminal units in the smart grid, in the future, it is inevitable that the deep integration of smart home and SG becomes a development trend whose direction is mainly reflected in three aspects. Firstly, in terms of smart home energy production and utilization, the openness of SG determines that clients have the dual attributes of consumers and producers of electric energy. With the development of SG, more and more smart homes will begin to install distributed energy equipment and use microgrid technology for flexible control, forming an effective supplement to the grid and achieving a win-win for the grid and clients. Secondly, in aspects of energy consumption, the simultaneous management of household energy and information can be realized through refined demand-side management. Finally, when it comes to services, by realizing the real-time two-way interaction between the grid and home, it can guide clients to actively participate in the construction and management of SG and provide clients with better services [5].

However, when a wide variety of smart home applications with diametrically different functional segmentation are connected and controlled over network, there are many problems such as data privacy, security, limited bandwidth in trunk links, serious energy consumption, link congestion, etc., especially data security [6]. Generally smart home applications are intelligent independently, such as a smart security system and a surveillance camera typically, they rely on the cloud platform to achieve remote control, in the event of a network malfunction, the clients may lose control of the applications [7]. Moreover, the smart home does not enable entirely coordinated work of multiple smart devices in conjunction with each other [8]. However, the emergence of edge computing technology makes it possible for intelligent connectivity and stable operation among smart home applications.

Edge computing is also known as “Fog Computing”, which refers to inserting an intermediate node layer between user and cloud computing data center [9]. The intermediate node layer is closer to users in the network topology and located in the edge of the whole network [10]. As a supplement and continuation of cloud computing, edge computing has the characteristics of strong scalability, low latency, high mobility, bandwidth saving, low energy consumption, system security and high performance [11]. In recent years, edge computing has been applied into vehicle networking, HD (High Definition) video transmission, VR (Virtual Reality), AR (Augmented Reality) and other fields [12,13]. The edge nodes deployed in the client’s home collect information through wired/wireless sensors. The information includes indoor temperature, brightness, air humidity and image information collected by outdoor/indoor cameras for comprehensive analysis. Then the edge nodes issue specific commands to smart home applications to complete the intelligent linkage control of the client’s home lighting, temperature system and alarm mechanism.

**Figure 1:**
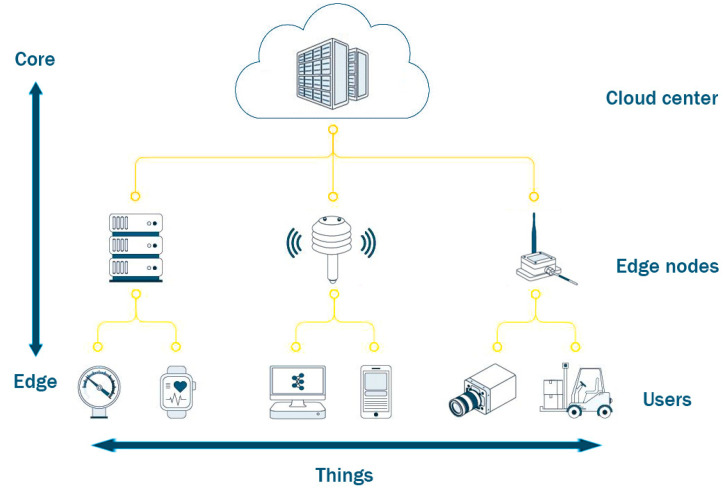
The conventional structure of edge computing.

According to network topology, the conventional structure of edge computing contains three layers: cloud layer, edge layer and user layer [14]. As shown in Figure 1, cloud layer refers to the remote large-scale cloud center, which provides necessary data processing support for smooth operation of fog layer. Due to its high energy consumption, large volume, cooling process and optimization of transmission nodes, cloud center is subject to many conditions in layout location. Edge layer, as the name suggests, is closer to the “edge” (user) than the “core” (cloud center). The edge layer contains a great number of edge nodes, which are located around users and act as small computing centers with the ability of processing tasks, storing data and connecting to the network [15]. The user layer refers to the terminal facilities that directly serve clients. It not only includes mobile phones, computers and other electronic devices, but also includes the terminals that continue to be endowed with wisdom under the general trend of IoT, such as smart air conditioners, sweeping robots, precision medical devices, et al. However, in a smart home environment, it is necessary for smart home applications to operate independently from cloud center due to link congestion, data security, time delay and privacy [16,17]. Thus, we design an edge computing resource allocation structure without using cloud center for this scenario. By allocating and provisioning resources among smart home applications, smart homes can get rid of the reliance on resource from cloud center. In fact, this structure of resource allocation has been applied in many fields [18]. For instance, the Unmanned Aerial Vehicle (UAV) flight data collection system stores the collected data in the Edge Data Center (EDC) in real time and immediately executes the data analysis algorithm without communicating with cloud center [19]. Then the control platform can flexibly adjust the task flow according to the analysis results.

At present, there are two mainstream resource allocation methods for edge computing without using cloud center [20]. One is from the perspective of users, in order to obtain the optimal resource allocation, the most important issue is to select the appropriate edge nodes to perform the calculation migration. This method assumes that all users are in a hotspot area that covers edge computing networks and can access multiple edge nodes nearby. Thus, the users take advantage of the game theory and operational research to select the best node and form optimal resource scheduling strategies [21]. The other is from the perspective of edge nodes. The key consideration is how to reasonably form edge node clusters to achieve the optimization of the overall performance of the system. This method believes that edge nodes need to complete resources through the collaborative work [22,23]. This paper attempts to take the participants, edge nodes and users (all smart home applications) of resource allocation into a market, and use the rules of market equilibrium to achieve optimal resource allocation, which can overcome the shortcomings of only considering one party in the process of resource allocation in the above research.

In this paper, we apply the resource pricing and the utility theory in economics to investigate the resource allocation and scheduling of edge computing without using cloud center for smart home environment. In order to make the modeling process simpler, we take bandwidth resources as an example to investigate the resource allocation of edge computing for smart homes. Although it is at present that the available bandwidth exceeds significantly the local application demands, we think that it is worth discussing the bandwidth allocation in smart home environment in the future. The reasons why we believe it are as follows. On the one hand, at the supply side, the last decade has witnessed an explosion of data traffic over the network attributed to the development of mobile communication. With the advent and extension of fifth-generation (5G) mobile networks, it is inevitable to emerge the situation of the exponential rise in data-demand and increasing demand in bandwidth. On the other hand, at the demand side, with the improvement of people’s quality of life and soaring demand for more convenient and comfortable services, it may appear in our life that a variety of IoT applications and smart homes including 4K/8K Ultra High Definition (UHD) video and virtual/augmented reality(VR/AR) require low-latency and high-bandwidth. We also divide the tasks generated by smart home applications into two types: the tasks with low requirement on latency and high requirement on processing capacity, and the tasks with high requirement on latency and low requirement on processing capacity. For the first type of tasks, we introduce the utility function aiming at optimizing resource transmission delay, while for the second type, we introduce the utility function aiming at optimizing resource transmission volume. Different utility functions are chosen to describe and maximize the characteristics of users when dealing with these two types of tasks. Based on the opinion of maximizing utility and the rules of market equilibrium, we propose the utility maximization model of resource allocation for different tasks of edge computing without using cloud center. Subsequently, we analyze the optimal resource allocation for the two types of tasks and obtain the optimum for each one. Then, we design the resource allocation algorithm by using the Lagrangian method and low-pass filtering theory which is assisted to eliminate oscillations and increase convergence speed. Finally, some numerical examples are given to verify the performance of the algorithm for two types of tasks.

## 2. Related Work

With the economic boom and technical advancement, an increasing number of smart devices find their way into people’s daily life, which have also received a lot of research attention from various communities like researchers, business and government. Lee et al. [24] designed a two-level Deep Reinforcement Learning (DRL) framework for optimal energy management of smart homes. In the proposed simulation, two agents (an air conditioner and a washing machine) interact with each other to schedule the optimal home energy consumption efficiently. Al-Ali et al. [25] presented an energy management system for smart homes and better meeting the needs of clients through big data analytics and business intelligence. Wang et al. [26] proposed a new task scheduling approach to fulfil the requirements of smart homes and healthcare regarding tight set situations of the elderly or patients, using edge computing-themed processing schemes with a focus on real-time issues. Procopiou et al. [27] proposed a lightweight detection algorithm for IoT devices based on chaos prediction and chaos theory–Chaos Algorithm (CA) which is used for the identification of Flooding and Distributed Denial of Service (DDoS) attacks to provide a secure network environment for IoT-based smart home systems. Yang et al. [28] proposed an IoT-based smart home security monitoring system that can enhance the security performance of system by improving the False Positive Rate (FPR) and reducing the network latency over the traditional security system.

At present, for the problem of resource allocation for edge computing without using cloud center, there are two main types of research approach. The first one is from the user’s perspective, Guo et al. [22] illustrated that it is an important method for achieving optimal resource allocation to filter out appropriate edge nodes to perform computational migration. The authors assumed that all users are within range of edge computing hotspots and have access to multiple edge servers. They proposed an optimal task scheduling strategy based on a discrete Markov decision process (MDP) framework to achieve optimal allocation of tasks and resources. Meanwhile, they also presented an index-based allocation strategy to reduce the computation complexity and communication overheads associated with the implementation of this policy. Finally, each user can find the most suitable edge server to minimize energy consumption and latency. The second one is from the edge node’s perspective, Queis et al. [23] analyzed the impact of cluster size (i.e., the number of edge nodes performing computational tasks) on service latency and energy consumption of edge nodes. Their research showed that increasing the number of edge nodes does not always reduce execution latency; If the transmission latency is longer than the computational latency at the edge nodes, the overall service latency may increase. Therefore, proper construction methods for edge node cluster and node selection methods play a crucial role in system performance. Queis et al. [29] proposed three different clustering strategies regarding optimization of service latency, overall energy consumption of the clusters and energy consumption of the nodes of the clusters in order to validate the impact of different clustering strategies on edge node cluster characteristics (size, latency, and energy consumption). Li et al. [30] proposed a self-similarity-based load balancing (SSLB) mechanism for large-scale fog computing which focuses on the applications processed on fog infrastructure. They also presented an adaptive threshold policy and corresponding scheduling algorithm, which guarantees the efficiency of SSLB. Queis et al. [31] designed a two-step method for implementing user task scheduling and resource allocation of nodes. The first step is local resource allocation, where each edge node allocates its resources to nearby users according to specific scheduling rules; The second step is the creation of a cluster of edge nodes for users that were not allocated compute resources in the first step. Sun et al. [32] proposed a two-level resource scheduling model, which includes resource scheduling among various fog clusters and resource scheduling among fog nodes in the same fog cluster. Besides, they believed that the edge layer and the terminal layer partially intersect because the mobile terminal devices in these intersections are not only fog resource requesters but also resource providers. 

Zhou et al. [33] proposed a cloud resource scheduling algorithm based on Markov prediction model which is put forward to solve the problem of task scheduling and load balancing in cloud service node failure situation, including the judgment of node load degree, the selection of migrated task and nodes, and the decision of migration routing. The goal is to achieve rapid cloud service recovery and to improve the reliability of cloud services. Zheng et al. [34] proposed an energy consumption optimization problem that considers latency performance. The model includes energy consumption and latency in the process of execution and transmission of local devices, fog nodes and cloud centers. Liu et al. [11] presented a joint model including consumption, latency, and payment costs in fog computing heterogeneous networks to solve a multi-objective optimization problem between nodes and users by using queueing theory and operations research. Wang et al. [35] designed an unloading system in real-time traffic management based on fog node-based IoV (Internet of Vehicle) system, which expands cloud computing by using parking and moving vehicles as fog nodes. 

In the research of resource allocation in the network, Li et al. [36] applied the hybrid cloud resource optimization model to the mobile client, and used a two-stage optimization process to provide a scheme for resource allocation of mobile client in mobile cloud environment. The first stage emphasizes that mobile cloud users achieve utility optimization under cost and energy constraints, and the second stage is that mobile cloud providers run multiple servers within the energy loss limit to perform the work of mobile cloud users. Li et al. [37] proposed a resource allocation model for elastic services based on utility optimization with the objective of maximizing network utility from the perspective of users’ utility optimization. In allocating bandwidth resources for migrating enterprise applications to the cloud, Li et al. [38] set the objective function to the problem of transmission time optimization, which makes the utility and satisfaction of enterprise users increase by minimizing cloud migration time. Nguyen et al. [39] elaborated a price-based edge computing method of resource allocation, which mainly expounds the multiple competitive service processes from heterogeneous edge nodes with limited ability to network edge under the efficient resource allocation framework of the market. In the process of explanation, the framework proved that the balanced allocation of resources realized Pareto optimization and met the fairness, proportionality and sharing incentives of allocation expectations.

## 3. Resource Allocation Model of Edge Computing without Using Cloud Center for Smart Homes

In a smart home environment, we assume that all smart home applications are composed of edge nodes and users, which mainly depends on whether their capacities meet task requirements. The tasks generated by the users in edge computing without using cloud center can only be solved at the edge nodes. Although the edge nodes are closer to the users in the topological position, when it comes to process different types of tasks, especially the tasks with large amount of calculation and storage, this paradigm has a massive number of limitations. In fact, there are large amounts of tasks that may appear in edge computing. We assume that these tasks of smart homes environment can be divided into two types: one is corresponding to the tasks with low requirement on latency and high requirement on processing capacity, and the other is associated with the tasks with high requirement on latency and low requirement on processing capacity. In this section, inspired by the concept of utility in economics, we formulate the resource allocation model of edge computing for smart home environment without using cloud center and adopt various utility strategies to process properly different types of tasks.

### 3.1. Model Description

**Figure 2:**
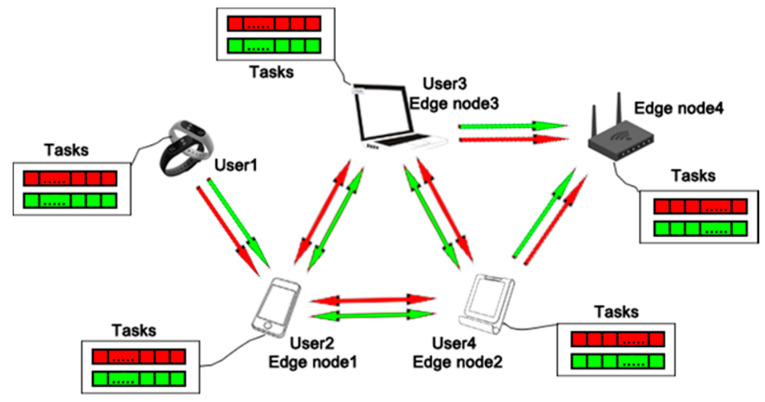
The structure of edge computing without using cloud center in smart home environment.

In the traditional edge computing structure, the remote cloud center, edge nodes and users form a close relationship through the networks. However, considering the data security and privacy of smart homes, avoiding the hosting costs incurred by uploading tasks to the cloud center and reducing the latency of smart home applications, we design a new edge computing structure for the smart home that does not require the involvement of cloud center. As shown in Figure 2, the user represents a smart home application whose capacity does not meet the task requirements and the edge node is a smart home application at the bottom of network structure that has spare computing power and provides the compute and storage resources for its surrounding users. The arrow direction represents the tasks generated by users that are processed by nearby edge nodes. Their spare computing power eliminates the need to transmit data to the cloud center and decentralizes processing power to ensure real-time processing with reduced latency, while diminishing bandwidth and network storage requirements. In fact, many smart home applications both act as users sending task requests to surrounding edge nodes and as edge nodes receiving and resolving tasks from surrounding users in a smart home environment. For example, a mobile phone can act as an edge node between a smart bracelet and a cloud center, processing service requests from the bracelet about exercise and health monitoring, and as a user generating tasks about games, videos, social networks, et al. However, the tasks generated by users can only be processed by the adjacent edge nodes in a smart home environment due to the lack of cloud center. For the purpose of processing various tasks in different strategies properly, the tasks generated by users or received by edge nodes are divided into two types. As shown in Figure 2, the red tasks represent the tasks with high requirement on latency and low requirement on processing capacity (For example, game commands in phone, shopping online by Taobao VR) and the green tasks represent the tasks with low requirement on latency and high requirement on processing capacity (For example, watching HD video on Smart TV). By adopting different processing strategies for the two kinds of tasks, the model can take advantage of the relationship of edge nodes and users to solve resource allocation in edge computing without the participation of cloud center.

### 3.2. Model Formulation

Due to security and privacy requirements, it is better to divide the entire network into multiple non-connected regions. In this paper, we focus on a smart home environment which should be regarded as an independent region to avoid the network intrusion and attack. Thus, we assume that all smart home applications are in a home (namely an independent region) and are not connected with cloud center, then they are considered in a resource allocation market. In the market, all nodes (smart home applications) are divided into customers (users) and resource providers (edge nodes) according to the contents of the previous section. In the following description, we use “resource provider/provider” instead of edge node and “resource consumer/consumer” instead of user. We take bandwidth resources as an example to intuitively analyze the resource allocation of edge computing in a smart home environment. Each customer sends a task processing request to one or more resource providers. Each resource provider receives requests from one or several resource customers and provides them with an appropriate amount of resources. In order to differentiate between resource providing nodes and resource requesting nodes in the resource allocation model and distinguish resource allocation requirements for different types of tasks, we introduce the set P of resource providers, the set R of resource customers for the tasks with high requirement on latency and low requirement on processing capacity and the set S of resource customers for the tasks with low requirement on latency and high requirement on processing capacity. A node is a member of P,R or S if it provides or requests at least one content of the two kinds of tasks, respectively. Of course, one node can be a member of all sets. Let  P(r) or P(s) be the set of providers offering resources for customers r∈R or s∈S. Thus, if a provider p∈P offers resources for customer r∈R or s∈S, then p∈P(r) or p∈P(s). Also, let S(p) or  R(p) be the set of customers that request resources from provider p for the two types of tasks, Thus, if customer s or r requests resources from provider p, then s∈S(p) or r∈R(p). Note that only when s∈S(p), r∈R(p), then p∈P(s), p∈P(r). 

Assume that the resource allocation between providers and customers in a smart home environment can be viewed as offering a certain amount of requested bandwidth. Each provider receives requests from customers and needs to serve the customers by providing with a certain amount of bandwidth. When processing a task with low requirement on latency and high requirement on processing capacity, denote the resources granted by provider p to customer s as $x_{ps}$, thus the aggregate resources granted by providers to customer s is $y_{s}=\underset{p:p\in P\left(s\right)}{\sum }x_{ps}$ and the resources provided by provider p to all its customers is $z_{p}^{L}=\underset{s:s\in S\left(p\right)}{\sum }x_{ps}$, the utility generated by the customer s is $U_{s}\left(y_{s}\right)$. Similarly, when processing a task with high requirement on latency and low requirement on processing capacity, denote the resources granted by provider p to customer r as $x_{pr}$, thus the aggregate resources granted by providers to customer r is $y_{r}=\underset{p:p\in P\left(r\right)}{\sum }x_{pr}$ and the resources provided by provider p to all its customers is $z_{p}^{H}=\underset{r:r\in R\left(p\right)}{\sum }x_{pr}$, the utility generated by the customer r is $U_{r}\left(y_{r}\right)$. Meanwhile, the resource allocation between providers and customers is subject to their capacity. Denote $C_{r}^{d}$, $C_{s}^{d}$ as the capacity of bandwidth resources of the downstream links of customers r and s, respectively. Denote $C_{p}^{uH}$, $C_{p}^{uL}$ as the capacity of reserved bandwidth resources of the upstream link of provider p for the tasks with high requirement on latency and the tasks with low requirement on latency, which are discussed in previous part. Therefore, for each provider  p, we can obtain that $z_{p}^{L}\leq C_{p}^{uL}$, which means that the total granted resources for customers requesting the tasks with low requirement on latency and high requirement on processing capacity do not exceed the reserved upstream link capacity $C_{p}^{uL}$. For each provider p, we also obtain that $z_{p}^{H}\leq C_{p}^{uH}$, which means that the total granted resource for customers requesting the tasks with high requirement on latency and low requirement on processing capacity do not exceed the reserved upstream link capacity $C_{p}^{uH}$. For each customer s, $y_{s}\leq C_{s}^{d}$, for each customer r, $y_{r}\leq C_{r}^{d}$, which mean that the total obtained bandwidth resources do not exceed the downstream link capacity of each customer.

This paper considers the aggregated utility of all resource customers, which can avoid deviations caused by different resource satisfaction utility of individual customers. Then the optimization problem of the edge computing resource allocation model without using cloud center can be modelled as following.

(1) $$Max\underset{s:s\in S}{\sum }U_{s}\left(y_{s}\right)+\underset{r:r\in R}{\sum }U_{r}\left(y_{r}\right)\;\;s.t\;\sum_{p:p\in P\left(s\right)}x_{ps}=y_{s},\forall s\in S\;\;\sum_{p:p\in P\left(r\right)}x_{pr}=y_{r},\;\forall r\in R\;\sum_{p:p\in P\left(s\right)}x_{ps}\leq C_{s}^{d},\forall s\in S\;\sum_{p:p\in P\left(r\right)}x_{pr}\leq C_{r}^{d},\;\forall r\in R\;\;\sum_{s:s\in S\left(p\right)}x_{ps}\leq C_{p}^{uL}\;\;\sum_{r:r\in R\left(p\right)}x_{pr}\leq C_{p}^{uH}\;\;Over\;x_{ps}\geq 0,x_{pr}\geq 0,C_{s}^{d},C_{r}^{d},C_{p}^{uL},C_{p}^{uH}\geq 0,\;s\in S,r\in R,p\in P$$

The following utility functions we use in this paper were described in the previous literatures. The Equation (2) is used to achieve a distinguished variant of utility-based fairness, which has drawn a great deal of attention and interest in recent years [40,41,42,43]. The Equation (3) is a variant of fairness, which can be reduced to the minimization of time delay and has also been receiving increasing attention [44,45].

(2) $$U_{s}\left(y_{s}\right)=\omega_{s}log\left(y_{s}+1\right)$$

(3) $$U_{r}\left(y_{r}\right)=\left(T_{r}-\frac{M_{r}}{y_{r}}\right)\times \frac{sgn\left(y_{r}-B_{r}\right)+1}{2}$$

In the above Equation (2), $\omega_{s}$ refers to the willingness to pay (WTP) of customer s for processing the tasks with low requirement on latency and high requirement on processing capacity [46,47]. In the above Equation (3). $M_{r}$ refers to the amount of task for customer r.$\;T_{r}$ refers to the time limit for customer r. $B_{r}$ refers to the minimum bandwidth requirement for customer r during resource allocation. When $y_{r}\leq B_{r}$, $\left(T_{r}-\frac{M_{r}}{y_{r}}\right)\times \frac{sgn\left(y_{r}-B_{r}\right)+1}{2}=0$, and when $y_{r}>B_{r}$, $\left(T_{r}-\frac{M_{r}}{y_{r}}\right)\times \frac{sgn\left(y_{r}-B_{r}\right)+1}{2}\neq 0\;$ [48].

The notations used in this paper are summarized in Table 1.

### 3.3. Model Analysis

In the previous section, we have established mathematical model of edge computing without using cloud center for resource allocation in a smart home environment. In this section, we analyze the resource allocation model and regard it as the original problem of the resource allocation model, then we give the Lagrangian function of this nonlinear optimization problem.

(4) $$\begin{array}{cc} L\left(x,y;\lambda ,\mu ,\nu ,\delta^{2}\right) & =\underset{s:s\in S}{\sum }\omega_{s}\log\left(y_{s}+1\right)+\underset{r:r\in R}{\sum }\left[\left(T_{r}-\frac{M_{r}}{y_{r}}\right)\times \left(\frac{sgn\left(y_{r}-B_{r}\right)+1}{2}\right)\right]\\ & +\underset{s:s\in S}{\sum }\lambda_{s}\left(\underset{p:p\in P\left(s\right)}{\sum }x_{ps}-y_{s}\right)+\underset{s:s\in S}{\sum }\nu_{s}\left(C_{s}^{d}-\underset{p:p\in P\left(s\right)}{\sum }x_{ps}-\delta^{2}{}_{s}\right)\\ & +\underset{r:r\in R}{\sum }\lambda_{r}\left(\underset{p:p\in P\left(r\right)}{\sum }x_{pr}-y_{r}\right)\\ & +\underset{r:r\in R}{\sum }\nu_{r}\left(C_{r}^{d}-\underset{p:p\in P\left(r\right)}{\sum }x_{pr}-\delta^{2}{}_{r}\right)\\ & +\underset{p:p\in P}{\sum }\mu_{p}^{L}\left(C_{p}^{uL}-\underset{p:p\in P\left(s\right)}{\sum }x_{ps}-\delta^{2}{}_{ps}\right)\\ & +\underset{p:p\in P}{\sum }\mu_{p}^{H}\left(C_{p}^{uH}-\underset{r:r\in R\left(p\right)}{\sum }x_{pr}-\delta^{2}{}_{pr}\right) \end{array}$$

where λ is the price vector of elements $\lambda_{s},\lambda_{r}\geq 0$, which can be considered as the price per unit bandwidth paid by customer s and r, respectively; μ is the price vector of elements $\mu_{p}^{L},\mu_{p}^{H}\geq 0$, which can be considered as the price per unit bandwidth charged by the upload link of provider p for the two types of tasks; ν is the price vector of elements $\nu_{s},\nu_{r}\geq 0$, which can be considered as the price paid by the download link of customer s and r, respectively. $\delta^{2}$ is regarded as the slack variable.

(5) $$\begin{array}{cc} L\left(x,y;\lambda ,\mu ,\nu ,\delta^{2}\right) & =\underset{s:s\in S}{\sum }[\omega_{s}\log\left(y_{s}+1\right)-\lambda_{s}y_{s}]\\ & +\underset{r:r\in R}{\sum }\left[\left(T_{r}-\frac{M_{r}}{y_{r}}\right)\times \left(\frac{sgn\left(y_{r}-B_{r}\right)+1}{2}\right)-\lambda_{r}y_{r}\right]\\ & +\underset{s:s\in S}{\sum }\underset{p:p\in P\left(s\right)}{\sum }x_{ps}\left(\lambda_{s}-\nu_{s}-\mu_{ps}\right)+\underset{r:r\in R}{\sum }\underset{p:p\in P\left(r\right)}{\sum }x_{pr}\left(\lambda_{r}-\nu_{r}-\mu_{pr}\right)\\ & +\underset{s:s\in S}{\sum }\nu_{s}\left(C_{s}^{d}-\delta^{2}{}_{s}\right)+\underset{r:r\in R}{\sum }\nu_{r}\left(C_{r}^{d}-\delta^{2}{}_{r}\right)+\underset{p:p\in P\left(s\right)}{\sum }\mu_{p}^{L}\left(C_{p}^{uL}-\delta^{2}{}_{ps}\right)\\ & +\underset{p:p\in P\left(r\right)}{\sum }\mu_{p}^{H}\left(C_{p}^{uH}-\delta^{2}{}_{pr}\right) \end{array}$$

The first two terms in the above equation take $y_{s}$ and $y_{r}$ as independent variables, and the third and fourth terms take $x_{ps}$ and $x_{pr}$ as independent variables. The objective function of the dual problem can be written as

(6) $$\begin{array}{cc} D\left(\lambda ,\mu ,\nu ,\delta^{2}\right) & =\max_{x,y}\;L\left(x,y;\lambda ,\mu ,\nu ,\delta^{2}\right)\\ & =\underset{s:s\in S}{\sum }\varphi_{s}\left(\lambda_{s}\right)+\underset{r:r\in R}{\sum }\varphi_{r}\left(\lambda_{r}\right)+\underset{s:s\in S}{\sum }\underset{p:p\in P\left(s\right)}{\sum }\varsigma_{ps}\left(\lambda_{s},\nu_{s},\mu_{p}^{L}\right)\\ & +\underset{r:r\in R}{\sum }\underset{p:p\in P\left(r\right)}{\sum }\varsigma_{pr}\left(\lambda_{r},\nu_{r},\mu_{p}^{H}\right)+\underset{s:s\in S}{\sum }\nu_{s}\left(C_{s}^{d}-\delta^{2}{}_{s}\right)+\underset{r:r\in R}{\sum }\nu_{r}\left(C_{r}^{d}-\delta^{2}{}_{r}\right)\\ & +\underset{p:p\in P\left(s\right)}{\sum }\mu_{p}^{L}\left(C_{p}^{uL}-\delta^{2}{}_{ps}\right)+\underset{p:p\in P\left(r\right)}{\sum }\mu_{p}^{H}\left(C_{p}^{uH}-\delta^{2}{}_{pr}\right) \end{array}$$

From the above Equation (6) we can derive

(7) $$\varphi_{s}\left(\lambda_{s}\right)=\underset{y_{s}}{\max}U_{s}\left(y_{s}\right)-\lambda_{s}y_{s}$$

(8) $$\varphi_{r}\left(\lambda_{r}\right)=\underset{y_{r}}{\max}U_{r}\left(y_{r}\right)-\lambda_{r}y_{r}$$

(9) $$\varsigma_{ps}\left(\lambda_{s},\nu_{s},\mu_{p}^{L}\right)=\underset{x_{ps}}{\max}x_{ps}\left(\lambda_{s}-\nu_{s}-\mu_{p}^{L}\right)$$

(10) $$\varsigma_{pr}\left(\lambda_{r},\nu_{r},\mu_{p}^{H}\right)=\underset{x_{pr}}{\max}x_{pr}\left(\lambda_{r}-\nu_{r}-\mu_{p}^{H}\right)$$

Then the optimal resource allocation for tasks with low requirement on latency and high requirement on processing capacity can be expressed as

(11) $$y_{s}^{*}\left(\lambda_{s}\right)=argmax\;\;U_{s}\left(y_{s}\right)-\lambda_{s}y_{s}$$

(12) $$x_{ps}^{*}\left(\nu_{s},\mu_{p}\right)=argmax\;x_{ps}\left(\lambda_{s}\right)\left(\lambda_{s}-\nu_{s}-\mu_{p}^{L}\right)$$

where $\sum_{p:p\in P\left(s\right)}x_{ps}\left(\lambda_{s}\right)=y_{s}^{*}\left(\lambda_{s}\right)$.

The optimal resource allocation for tasks with high requirement on latency and low requirement on processing capacity can be expressed as

(13) $$y_{r}^{*}\left(\lambda_{r}\right)=argmax_{\;}\;U_{r}\left(y_{r}\right)-\lambda_{r}y_{r}$$

(14) $$x_{pr}^{*}\left(\nu_{r},\mu_{p}\right)=argmax\;x_{pr}\left(\lambda_{r}\right)\left(\lambda_{r}-\nu_{r}-\mu_{p}^{H}\right)$$

where $\sum_{p:p\in P\left(r\right)}x_{pr}\left(\lambda_{r}\right)=y_{r}^{*}\left(\lambda_{r}\right)$.

From the perspective of resource customer, customers s and r are trying to maximize their own utilities, which are determined by the allocation bandwidth resource $y_{s}$, $y_{r}$ they obtain. The customers s and r must pay for bandwidth resource they use, $\lambda_{s}y_{s},\lambda_{r}y_{r}$ are the cost that customers s and r are willing to pay, respectively. $\nu_{s}$,$\;\nu_{r}$ are the prices charged by the download links of customers s and r, so $x_{ps}\nu_{s}$,$\;x_{pr}\nu_{r}$ are the costs charged by the download links of customers s and r. From the perspective of resource provider, each provider p is trying to maximize its revenue. $\mu_{p}^{L},\;\mu_{p}^{H}$ are the prices per unit of bandwidth charged by the upload link of provider p for the two types of different tasks, so $x_{ps}\mu_{p}^{H}$, $x_{pr}\mu_{p}^{L}$ are the costs charged by the upload link of provider p for the two types of tasks.

Thus, the dual problem of resource allocation model is

(15) $$min\;\;\;\;\;\;D\left(\lambda ,\nu ,\mu_{\;}\right)\;over\;\;\;\;\;\;\;\lambda_{s}\geq 0,\lambda_{r}\geq 0,\nu_{s}\geq 0,\nu_{r}\geq 0,\mu_{p}^{H}\geq 0,\mu_{p}^{L}\geq 0,s\in S,r\in R,p\in P$$

The objective of the dual problem is to minimize the total cost of transmission for all nodes while ensuring a certain level of satisfaction for customers.

**Theorem** **1.** *The utility function is concave with respect to the variables for both types of tasks, so the optimal resource allocation for each customer and the optimal objective value of the original problem can be obtained, and the optimal objective value of the original problem equals to the optimal objective value of the dual problem, i.e.,* $\;U^{*}=D^{*}$ *, but the optimal resource allocation provided by each provider, i.e.,* $x_{ps}^{*}$ *,* $x_{pr}^{*}$ *, is not unique.*

**Proof** **of** **Theorem 1.** From the convex optimization theory [49], since the objective function $\sum_{s:s\in S}U_{s}\left(y_{s}\right)+\sum_{r:r\in R}U_{r}\left(y_{r}\right)$ for both types of tasks is concave with respect to its variables and the set of constraints in the resource allocation model is convex, thus the resource allocation model is a convex optimization problem. Then the optimal resource allocation can be obtained by applying the convex optimization approach. The optimal total resource allocation for each customer, i.e., $y_{s}$, $y_{r}$, is unique. But the optimal resource provided by each provider, i.e., $x_{ps}^{*}$, $x_{pr}^{*}$ may not be unique. The result is obtained. □

**Theorem** **2.** *When the optimum of the resource allocation model is achieved, if two providers simultaneously provide resource to a customer, then the prices charged by these two providers are equal. This result also holds when considering resource customers for other different types of tasks.*

**Proof** **of** **Theorem 2.** 1. If customers receive tasks with low requirement on latency and high requirement on processing capacity, in the case where the optimum of the resource allocation model is achieved, we can take advantage of the Karush-Kuhn-Tucker (KKT) conditions to obtain that

$$\frac{\omega_{s}}{y_{s}^{*}+1}-\lambda_{s}^{*}=0,y_{s}^{*}>0,\forall s:s\in S\;\lambda_{s}^{*}-\nu_{s}^{*}-\mu_{p}^{L*}=0,x_{ps}^{*}>0,\forall s:s\in S\;,\forall p:p\in P$$

The above equation is a necessary condition for the existence of an optimal solution to the resource allocation problem. For two providers that offer resources to the same customer, e.g., $p_{1},p_{2}\in P\left(s\right)$*,* the optimal prices charged by these two providers are equal, i.e.,

$$\mu_{p_{1}}^{L*}=\mu_{p_{2}}^{L*}=\lambda_{s}^{*}-\nu_{s}^{*}$$

2. If the customers receive tasks with high requirement on latency and low requirement on processing capacity, in the case where the optimum of the resource allocation model is achieved, we can take advantage of the KKT conditions to obtain that

$$\frac{M_{r}}{{\left(y_{r}^{*}\right)}^{2}}-\lambda_{r}^{*}=0,y_{r}^{*}>0,\forall r:r\in R\;\lambda_{r}^{*}-\nu_{r}^{*}-\mu_{p}^{H*}=0,x_{pr}^{*}>0,\forall r:r\in R,\forall p:p\in P$$

The above equation is a necessary condition for the existence of an optimal solution to the resource allocation problem. For two providers that provide resources to the same customer, e.g., $p_{1},p_{2}\in P\left(r\right)$, the optimal prices charged by these two providers are equal, i.e.,

$$\mu_{p_{1}}^{H*}=\mu_{p_{2}}^{H*}=\lambda_{r}^{*}-\nu_{r}^{*}$$

The result is obtained. □

## 4. Optimal Resource Allocation for Smart Homes

To maximize the aggregated utility of the resource allocation model in the smart home environment, we need to maximize the aggregated utility of the model for processing the tasks with high requirement on latency and low requirement on processing capacity and the aggregated utility of the model for processing the tasks with low requirement on latency and high requirement on processing capacity, respectively. The upload link of resource provider is often regarded as scarce resource and the corresponding constraints in the model are always active constraints. In this section, we assume that only the constraints of providers when uploading resources are considered in the process of analyzing optimal resource allocation. However, it is also critical that the amount of bandwidth resource received does not exceed the limit of the download link. In the Lagrangian function, $\delta^{2}$ refers to the remaining bandwidth resource of the participant in the resource allocation. Obviously, according to the KKT conditions when $\delta^{2}=0$, the constraint on the bandwidth resource of participant becomes active. When $\delta^{2}>0$, the constraint on the bandwidth resource of participant becomes non-active. In the following analysis, we assume that $\delta^{2}=0$, which means the remaining resources of provider and the spare capacity of download link are gone. Otherwise, the non-active constraints can be omitted and only active constraints are considered [50]. 

The utility functions are substituted into the resource allocation model for analyzing respectively. The model for processing the tasks with low requirement on latency and high requirement on processing capacity is

(16) $$max\underset{s:s\in S}{\sum }\omega_{s}log\left(y_{s}+1\right)\;s.t.\;\sum_{p:p\in P\left(s\right)}x_{ps}=y_{s},\forall s\in S\;\sum_{p:p\in P\left(s\right)}x_{ps}\leq C_{s}^{d},\forall s\in S\;\sum_{s:s\in S\left(p\right)}x_{ps}\leq C_{p}^{uL}\;\forall p\in P\;x_{ps}\geq 0,s\in S,r\in R,p\in P$$

The model for processing the tasks with high requirement on latency and low requirement on processing capacity is

(17) $$max\underset{r:r\in R}{\sum }\left(T_{r}-\frac{M_{r}}{y_{r}}\right)\times \left[\frac{sgn\left(y_{r}-B_{r}\right)+1}{2}\right]\;s.t.\;\sum_{p:p\in P\left(r\right)}x_{pr}=y_{r}\;\forall r\in R\;\sum_{p:p\in P\left(r\right)}x_{pr}\leq C_{r}^{d},\;\forall r\in R\;\sum_{r:r\in R\left(p\right)}x_{pr}\leq C_{p}^{uH}\;,\forall p\in P\;x_{pr}\geq 0,s\in S,r\in R,p\in P$$

### 4.1. The Optimal Resource Allocation for Customer s

We first don’t consider the download bandwidth constraint (i.e., the first inequality constraint) in the resource allocation model (16), and obtain the following Lagrangian function:

(18) $$L_{1}\left(x,y;\lambda ,\mu \right)=\underset{s:s\in S}{\sum }\omega_{s}\log\left(y_{s}+1\right)+\underset{s:s\in S}{\sum }\lambda_{s}\left(\underset{p:p\in P\left(s\right)}{\sum }x_{ps}-y_{s}\right)+\underset{p:p\in P\left(s\right)}{\sum }\mu_{p}^{L}\left(C_{p}^{uL}-\underset{s:s\in S\left(p\right)}{\sum }x_{ps}\right)$$

The Lagrangian function can be rewritten as

(19) $$L_{1}\left(x,y;\lambda ,\mu \right)=\underset{s:s\in S}{\sum }\left[\omega_{s}\log\left(y_{s}+1\right)-\lambda_{s}y_{s}\right]+\underset{s:s\in S}{\sum }\underset{p:p\in P\left(s\right)}{\sum }x_{ps}\left(\lambda_{s}-\mu_{p}^{L}\right)+\underset{p:p\in P\left(s\right)}{\sum }\mu_{p}^{L}C_{p}^{uL}$$

According to Equation (7), there is an optimal allocation

(20) $$y_{s}^{*}=U^{’}{}_{s}^{-1}\left(\lambda_{s}\right)=\frac{\omega_{s}}{\lambda_{s}^{*}}-1$$

Then, substituting (20) into (19), we obtain

(21) $$\bar{L_{1}}\left(x;\lambda ,\mu \right)=\underset{s:s\in S}{\sum }\left[\omega_{s}\log\left(\frac{\omega_{s}}{\lambda_{s}^{*}}\right)-\omega_{s}+\lambda_{s}^{*}+\lambda_{s}^{*}\underset{p:p\in P\left(s\right)}{\sum }x_{ps}\right]-\underset{s:s\in S}{\sum }\underset{p:p\in P\left(s\right)}{\sum }x_{ps}\mu_{p}^{L}+\underset{p:p\in P\left(s\right)}{\sum }\mu_{p}^{L}C_{p}^{uL}$$

Let $\partial \bar{L_{1}}\left(x;\lambda ,\mu \right)/\partial \lambda_{s}=0$, then we obtain the optimal price paid by customer s

$$-\frac{\omega_{s}}{\lambda_{s}}+1+\underset{p:p\in P\left(s\right)}{\sum }x_{ps}=0$$

(22) $$\lambda_{s}^{*}=\frac{\omega_{s}}{1+\sum_{p:p\in P\left(s\right)}x_{ps}}$$

Substitute the obtained result (22) into function (19) again

(23) $$\begin{array}{l} \bar{L_{1}}\left(x;\nu \right)=\underset{s:s\in S}{\sum }[\omega_{s}\log\left(1+\underset{p:p\in P\left(s\right)}{\sum }x_{ps}\right)-\omega_{s}+\left(\frac{\omega_{s}}{1+\sum_{p:p\in P\left(s\right)}x_{ps}}+1\right)\\ \;+\left(\frac{\omega_{s}}{1+\sum_{p:p\in P\left(s\right)}x_{ps}}+1\right)\times \underset{p:p\in P\left(s\right)}{\sum }x_{ps}]-\underset{s:s\in S}{\sum }\underset{p:p\in P\left(s\right)}{\sum }x_{ps}\mu_{p}^{L}\\ \;+\underset{p:p\in P\left(s\right)}{\sum }\mu_{p}^{L}C_{p}^{uL} \end{array}$$

Let $\partial \bar{L_{1}}\left(x;\nu \right)/\partial x_{ps}=0$, then we obtain the optimal price charged by provider p

$$\frac{\omega_{s}}{1+\sum_{p:p\in P\left(s\right)}x_{ps}}-\mu_{ps}=0$$

(24) $$\mu_{p}^{L*}=\frac{\omega_{s}}{1+\sum_{p:p\in P\left(s\right)}x_{ps}}$$

Therefore, when the download bandwidth constraint of each customer is not considered, we obtain $\mu_{p_{1}}^{L*}=\mu_{p_{2}}^{L*}=\lambda_{s}^{*}$, $p_{1},p_{2}\in P\left(s\right)$, s:s∈S. In this case, the price paid by the resource customer s for per unit bandwidth is equal to the price charged by the provider p for per unit bandwidth.

We assume that the entire network is divided into τ non-connected regions, where each region is composed of a sub-set of all nodes. According to Theorem 2, the optimal prices charged by resource providers in each region are equal, and they are all equal to the price paid by any customer s, $\mu_{p}^{L*}=\mu_{\tau }^{\;}$, $\lambda_{s}^{*}=\lambda_{\tau }^{\;}$, $\mu_{\tau }^{\;}=\lambda_{\tau }^{\;}$, where $s:s\in S_{\tau }$. $S_{\tau }$ refers to the collection of all resource consumers in the region. Particularly, when τ=1,  then $P_{\tau }=P,\;\mu_{\tau }^{\;}=\mu_{,\;}^{\;}\;S_{\tau }=S$.

Therefore, the transformed Lagrangian function can be expressed as

(25) $$\begin{array}{c} \bar{L_{1}}\left(x;\nu \right)=\underset{\tau }{\sum }\left[\underset{s:s\in S_{\tau }}{\sum }\omega_{s}\log\left(\underset{p:p\in p_{\tau }\left(s\right)}{\sum }x_{ps}+1\right)+\underset{p:p\in P_{\tau }}{\sum }\mu_{ps}^{\;}\left(C_{p}^{uL}-\underset{s:s\in S_{\tau }\left(p\right)}{\sum }x_{ps}\right)\right]\\ =\underset{\tau }{\sum }\left[\underset{s:s\in S_{\tau }}{\sum }\left(\omega_{s}\log\frac{\omega_{s}}{\nu_{\tau }^{\;}}-\omega_{s}+\mu_{\tau }^{\;}\right)+\underset{p:p\in P_{\tau }}{\sum }\mu_{\tau }^{\;}C_{p}^{uL}\right] \end{array}$$

where $\mu_{p}^{L*}=\mu_{\tau }^{\;}$, $\forall \;p\in P_{\tau }$.

Let $d\bar{L_{1}}\left(\mu_{\tau }^{\;}\right)/d\mu_{\tau }^{\;}=0$, then

$$\underset{\tau }{\sum }\left\{\underset{s:s\in S_{\tau }}{\sum }\left[\omega_{s}\times \frac{1}{\frac{\omega_{s}}{\mu_{\tau }^{\;}}}\times \frac{\omega_{s}}{-\left(\mu_{\tau }^{\;}{}^{2}\right)}+1\right]+\underset{p:p\in P_{\tau }}{\sum }C_{p}^{uL}\right\}=0$$

(26) $$\mu_{\tau }^{\;}=\frac{\sum_{s:s\in S_{\tau }}\omega_{s}}{\left|S_{\tau }\right|+\sum_{p:p\in P_{\tau }}C_{p}^{uL}}$$

Substituting the above result (26) into (24)

(27) $$y_{s}^{*}=\frac{\omega_{s}\left(\left|S_{\tau }\right|+\sum_{p:p\in P_{\tau }}C_{p}^{uL}\right)}{\sum_{s:s\in S_{\tau }}\omega_{s}}-1$$

$\left|S_{\tau }\right|$ indicates the number of customers who need resources in sub-region τ.

Recall that the optimal resource allocation of customer s is also subject to its download bandwidth capacity $C_{s}^{d}$, thus the optimal resource allocation $y_{s}^{*}$ has the following value

(28) $$\dot{y_{s}^{*}}=min\left\{C_{s}^{d},\frac{\omega_{s}\left(\left|S_{\tau }\right|+\sum_{p:p\in P_{\tau }}C_{p}^{uL}\right)}{\sum_{s:s\in S_{\tau }}\omega_{s}}-1\right\}$$

### 4.2. The Optimal Resource Allocation for Customer r

Following the analysis method for resource allocation (16), we obtain the Lagrangian function for resource allocation (17) as follows.

(29) $$\begin{array}{c} L_{2}\left(x,y;\lambda ,\mu \right)=\underset{r:r\in R}{\sum }\left\{\left(T_{r}-\frac{M_{r}}{y_{r}}\right)\times \left[\frac{sgn\left(y_{r}-B_{r}\right)+1}{2}\right]\right\}+\underset{r:r\in R}{\sum }\lambda_{r}\left(\underset{p:p\in P\left(r\right)}{\sum }x_{pr}-y_{r}\right)\\ +\underset{p:p\in P}{\sum }\mu_{p}^{H}\left(C_{p}^{uH}-\underset{r:r\in R\left(p\right)}{\sum }x_{pr}\right) \end{array}$$

The Lagrangian function can be rewritten as

(30) $$\begin{array}{c} L_{2}\left(x,y;\lambda ,\mu \right)=\underset{r:r\in R}{\sum }\left\{\left(T_{r}-\frac{M_{r}}{y_{r}}\right)\times \left[\frac{sgn\left(y_{r}-B_{r}\right)+1}{2}\right]-\lambda_{r}y_{r}\right\}\\ +\underset{r:r\in R}{\sum }\underset{p:p\in P\left(r\right)}{\sum }x_{pr}\left(\lambda_{r}-\mu_{p}^{H}{}_{\;}\right)+\underset{p:p\in P}{\sum }\mu_{p}^{H}{}_{\;}C_{p}^{uH} \end{array}$$

According to Equation (8), there is an optimal allocation

(31) $$y_{r}^{*}=U^{’}{}_{r}^{-1}\left(\lambda_{r}\right)={\left(\frac{M_{r}}{\lambda_{r}^{*}}\right)}^{\frac{1}{2}}$$

Then

(32) $$\bar{L_{2}}\left(x;\lambda ,\mu \right)=\underset{r:r\in R}{\sum }\left(T_{r}-2{\left(\lambda_{r}^{\;}M_{r}\right)}^{\frac{1}{2}}\right)+\underset{r:r\in R}{\sum }\underset{p:p\in P\left(r\right)}{\sum }x_{pr}\left(\lambda_{r}-\mu_{p}^{H}\right)+\underset{p:p\in P}{\sum }\mu_{p}^{H}{}_{\;}C_{p}^{uH}$$

Let $\partial \bar{L_{2}}\left(x;\lambda ,\mu \right)/\partial \lambda_{r}=0$, then we obtain the optimal price paid by user r.

$$-\frac{M_{r}}{{\left(\lambda_{r}^{\;}M_{r}\right)}^{\frac{1}{2}}}+\underset{p:p\in P\left(r\right)}{\sum }x_{pr}=0$$

(33) $$\lambda_{r}^{*}=\frac{M_{r}}{{\left(\sum_{p:p\in P\left(r\right)}x_{pr}\right)}^{2}}$$

Substitute the obtained result into function (31) again

(34) $$\begin{array}{c} \bar{L_{2}}\left(x;\mu \right)=\underset{r:r\in R}{\sum }\left[T_{r}-2{\left(\frac{M_{r}}{{\left(\sum_{p:p\in P\left(r\right)}x_{pr}\right)}^{2}}M_{r}\right)}^{\frac{1}{2}}\right]\\ +\underset{r:r\in R}{\sum }\underset{p:p\in P\left(r\right)}{\sum }x_{pr}\left[\frac{M_{r}}{{\left(\sum_{p:p\in P\left(r\right)}x_{pr}\right)}^{2}}-\mu_{p}^{H}\right]+\underset{p:p\in P}{\sum }\mu_{p}^{H}C_{p}^{uH} \end{array}$$

(35) $$\bar{L_{2}}\left(x;\mu \right)=\underset{r:r\in R}{\sum }\left(T_{r}-2\frac{M_{r}}{\sum_{p:p\in P\left(r\right)}x_{pr}}\right)+\underset{r:r\in R}{\sum }\left(\frac{M_{r}}{\sum_{p:p\in P\left(r\right)}x_{pr}}-\underset{p:p\in P\left(r\right)}{\sum }x_{pr}\mu_{p}^{H}{}_{\;}\right)+\underset{p:p\in P}{\sum }\mu_{p}^{H}C_{p}^{uH}$$

Let $\partial \bar{L_{2}}\left(x;\mu \right)/\partial x_{pr}=0$, then we obtain the optimal price charged by edge node p

(36) $$\mu_{p}^{H*}=\frac{M_{r}}{{\left(\sum_{p:p\in P\left(r\right)}x_{pr}\right)}^{2}}$$

On the basis of the above discussion, it can be concluded that when the optimal resource allocation is achieved, the following condition holds: $\mu_{p_{1}}^{H*}=\mu_{p_{1}}^{H*}=\lambda_{r}^{*},\;p_{1},p_{2}\in P\left(s\right),\;r:r\in R$.

We assume that the entire network is divided into γ non-connected regions, where each region corresponds to a part of nodes. According to Theorem 2, the optimal prices charged by resource providers in each region are equal, and they are all equal to the price paid by any customer r, $\mu_{p}^{H*}=\mu_{\gamma }^{\;}$, $\lambda_{r}^{*}=\lambda_{\gamma }^{\;}$, $\mu_{\gamma }^{\;}=\lambda_{\gamma }^{\;}$, where $r:r\in R_{\gamma }$, $p:p\in P_{\gamma }$. $R_{\gamma }$ refers to the collection of all resource consumers r in the region. Particularly, when γ=1,  then $R_{\gamma }=R,\;\mu_{\gamma }^{\;}=\mu_{p}^{H},\;P_{\gamma }=P$.

Therefore, the transformed Lagrangian function can be expressed as

(37) $$\begin{array}{cc} \bar{L_{2}}\left(x;\mu \right)=\underset{\gamma }{\sum } & \left[\underset{r:r\in R_{\gamma }}{\sum }\left(T_{r}-\frac{M_{r}}{\sum_{p:p\in P\left(r\right)}x_{pr}}\right)-\underset{r:r\in R\left(r\right)}{\sum }\underset{p:p\in P\left(r\right)}{\sum }x_{pr}\mu_{\gamma }+\underset{p:p\in P}{\sum }\mu_{\gamma }^{\;}{}_{\;}C_{p}^{uH}\right]\\ & =\underset{\gamma }{\sum }\{\underset{r:r\in R_{\gamma }}{\sum }\left[T_{r}-\frac{M_{r}}{{\left(\frac{M_{r}}{\mu_{\gamma }^{\;}}\right)}^{\frac{1}{2}}}-\mu_{\gamma }^{\;}\times {\left(\frac{M_{r}}{\mu_{\gamma }^{\;}}\right)}^{\frac{1}{2}}\right]+\underset{p:p\in P}{\sum }\mu_{\gamma }^{\;}{}_{\;}C_{p}^{uH}\}\\ & =\underset{\gamma }{\sum }\left\{\underset{r:r\in R_{\gamma }}{\sum }\left[T_{r}-2{\left(M_{r}\mu_{\gamma }^{\;}\right)}^{\frac{1}{2}}\right]+\underset{p:p\in P}{\sum }\mu_{\gamma }^{\;}{}_{\;}C_{p}^{uH}\right\} \end{array}$$

where $\mu_{p}^{H*}=\mu_{\gamma }^{\;}$, $r:r\in S_{\gamma }$.

Let $\bar{dL_{2}}\left(x;\mu_{pr}^{\;}\right)/d\mu_{\gamma }^{\;}=0$, then

(38) $$\mu_{\gamma }^{\;}={\left[\frac{\sum_{r:r\in R_{\gamma }}{\left(M_{r}\right)}^{\frac{1}{2}}}{\sum_{p:p\in P}C_{p}^{uH}{}_{\;}}\right]}^{2}$$

Comparing (33) and (36), and substituting $\mu_{\gamma }^{\;}$ into the above Equation (33)

(39) $$y_{r}^{*}=U^{’}{}_{r}^{-1}\left(\lambda_{r}\right)=\frac{{\left(M_{r}\right)}^{\frac{1}{2}}\sum_{p:p\in P}C_{p}^{uH}{}_{\;}}{\sum_{r:r\in R_{\gamma }}{\left(M_{r}\right)}^{\frac{1}{2}}}$$

Recall that the optimal resource allocation of customer r is also subject to its download bandwidth capacity $C_{r}^{d}$, thus the optimal resource allocation $y_{r}^{*}$ has the following value

(40) $$\dot{y_{r}^{*}}=min\left\{C_{r}^{d},\frac{{\left(M_{r}\right)}^{\frac{1}{2}}\sum_{p:p\in P}C_{p}^{uH}{}_{\;}}{\sum_{r:r\in R_{\gamma }}{\left(M_{r}\right)}^{\frac{1}{2}}}\right\}$$

## 5. Resource Allocation Algorithm for Smart Homes

### 5.1. Algorithm Introduction

In order to obtain the optimal resource allocation of edge computing without using cloud center in a smart home environment, this section will introduce a distributed algorithm that only depends on local information, which is in line with smart home. Since the objective of the resource allocation model is concave but not strictly concave, which results in the optimal resource allocation is usually not unique, so that the proposed algorithm may be oscillation. Thus, we introduce and apply the low-pass filtering method to eliminate the possible oscillation and improve the convergence speed [51]. We regard $\tilde{x_{ps}}\left(t\right),\tilde{x_{pr}}\left(t\right)$ as the optimal estimation of the $x_{ps}\left(t\right),x_{pr}\left(t\right)$ and summarize the detailed algorithm steps as follows

1. Each provider p updates the resources $x_{ps}\left(t\right),x_{pr}\left(t\right)$ for its customers s and r. The update rule for customer s is
  (41) $$x_{ps}\left(t+1\right)={\left\{\left(1-\theta_{ps}\right)x_{ps}\left(t\right)+\theta_{ps}\tilde{x_{ps}}\left(t\right)+\theta_{ps}\kappa_{ps}x_{ps}\left(t\right)\left[\lambda_{s}\left(t\right)-\nu_{s}\left(t\right)-\mu_{p}^{L}\left(t\right)\right]\right\}}_{x_{ps}\left(t\right)}^{+}$$
  (42) $$\tilde{x_{ps}}\left(t+1\right)=\left(1-\theta_{ps}\right)\tilde{x_{ps}}\left(t\right)+\theta_{ps}x_{ps}\left(t\right)$$
  (43) $$\lambda_{s}\left(t\right)=U_{s}^{\prime }\left(y_{s}\left(t\right)\right)$$
  (44) $$y_{s}\left(t\right)=\underset{p:p\in P\left(s\right)}{\sum }x_{ps}\left(t\right)$$
  The update rule for customer r is
  (45) $$x_{pr}\left(t+1\right)={\left\{\left(1-\theta_{pr}\right)x_{pr}\left(t\right)+\theta_{pr}\tilde{x_{pr}}\left(t\right)+\theta_{pr}\kappa_{pr}x_{pr}\left(t\right)\left[\lambda_{r}\left(t\right)-\nu_{r}\left(t\right)-\mu_{p}^{H}\left(t\right)\right]\right\}}_{x_{pr}\left(t\right)}^{+}$$
  (46) $$\tilde{x_{ps}}\left(t+1\right)=\left(1-\theta_{ps}\right)\tilde{x_{ps}}\left(t\right)+\theta_{ps}x_{ps}\left(t\right)$$
  (47) $$\lambda_{r}\left(t\right)=U_{r}^{\prime }\left(y_{r}\left(t\right)\right)$$
  (48) $$y_{r}\left(t\right)=\underset{p:p\in P\left(r\right)}{\sum }x_{pr}\left(t\right)$$
  where $\kappa_{ps},\kappa_{pr}>0$ are the small step sizes, $\theta_{ps},\theta_{pr}>0$ are the parameters for low-pass filtering method. $a={\left(b\right)}_{c}^{+}$ means c>0,a=b;c=0,a=max{0,b}.
2. Resource customers r,s update their prices $\nu_{s}\left(t\right),\nu_{r}\left(t\right)$ as the following law
  (49) $$\nu_{s}\left(t+1\right)={\left[\nu_{s}\left(t\right)+\alpha_{s}\frac{y_{s}\left(t\right)-C_{s}^{d}}{C_{s}^{d}}\right]}_{v_{s}\left(t\right)}^{+}$$
  (50) $$y_{s}\left(t\right)=\underset{p:p\in P\left(s\right)}{\sum }x_{ps}\left(t\right)$$
  (51) $$\nu_{r}\left(t+1\right)={\left[\nu_{r}\left(t\right)+\alpha_{r}\frac{y_{r}\left(t\right)-C_{r}^{d}}{C_{r}^{d}}\right]}_{v_{r}\left(t\right)}^{+}$$
  (52) $$y_{r}\left(t\right)=\underset{p:p\in P\left(r\right)}{\sum }x_{pr}\left(t\right)$$
  where $\alpha_{s},\alpha_{r}>0$ are the small step sizes.
3. Each resource provider p (edge node) updates its prices $\mu_{p}^{L}\left(t\right),\mu_{p}^{H}\left(t\right)$ by using the following method.
  (53) $$\mu_{p}^{L}\left(t+1\right)={\left[\mu_{p}^{L}\left(t\right)+\beta_{s}\frac{z_{p}^{L}-C_{p}^{uL}}{C_{p}^{uL}}\right]}_{\mu_{p}^{L}\left(t\right)}^{+}$$
  (54) $$z_{p}^{L}\left(t\right)=\underset{s:s\in S\left(p\right)}{\sum }x_{ps}\left(t\right)$$
  (55) $$\mu_{p}^{H}\left(t+1\right)={\left[\mu_{p}^{H}\left(t\right)+\beta_{r}\frac{z_{p}^{H}-C_{p}^{uH}}{C_{p}^{uH}}\right]}_{\mu_{p}^{H}\left(t\right)}^{+}$$
  (56) $$z_{p}^{H}\left(t\right)=\underset{r:r\in R\left(p\right)}{\sum }x_{pr}\left(t\right)$$
  where $\beta_{s},\beta_{r}>0$ are the small step sizes.

### 5.2. Basic Steps

According to the above-mentioned low-pass filtering approach, the augmented variables $\tilde{x_{ps}}\left(t\right),\tilde{x_{pr}}\left(t\right)$ can only eliminate the possible oscillation of the algorithm and improve the convergence speed, but it will not change the final optimum of the algorithm. At the optimal resource allocation, we obtain that $x_{ps}^{*}\left(t\right)=\tilde{x_{ps}^{*}}\left(t\right),x_{pr}^{*}\left(t\right)=\tilde{x_{pr}^{*}}\left(t\right)$. 

The iterative step size selected in the algorithm has a great influence on the convergence speed. The step size selected in the algorithm should be small enough to ensure convergence, but not too small, which causes the convergence speed to become too slow, so it is necessary to choose a suitable iteration step size. The algorithm can be iterated to the optimal resource allocation within reasonable convergence times.

The basic steps of the algorithm iteration procedure are described as following.

STEP 1: Initialize the variables and parameters.

We need to initialize the iteration step sizes $\kappa_{ps},\kappa_{pr},\alpha_{s},\alpha_{r},\beta_{s},\beta_{r}$ and initialize the bandwidth resource allocation $x_{ps}\left(t\right),x_{pr}\left(t\right)$ from provider p to customers s and r at time t.

STEP 2: Calculate the prices paid by customers s and r at time t.

Customers s and r calculate the offered bandwidth resources $y_{s}\left(t\right),y_{r}\left(t\right)$ that they receive based on the amount of bandwidth resources provided by providers at time t, and then calculate the prices they should pay for provider  p, respectively.

STEP 3: Calculate the prices charged by the upload and download links of each node at time t.

Provider p updates the amount of resources it provides for customers s and r at time t, $z_{p}^{L}\left(t\right)$ and $z_{p}^{H}\left(t\right)$. At the same time, provider p calculates the price $\mu_{p}\left(t+1\right)$ charged by the uploading link. Customers s and r update the prices $\nu_{s}\left(t+1\right),\nu_{r}\left(t+1\right)$ charged by their downloading links.

STEP 4: Update the bandwidth allocation of provider p at time t.

Provider p updates its resource allocation $x_{ps}\left(t+1\right),x_{pr}\left(t+1\right)$ for customers s and r at time t.

STEP 5: Set the stop condition.

The optimization problem considered in this paper is a convex optimization problem, which means that the optimal solution exists and is also the global optimal solution [52]. Therefore, when the algorithm reaches an equilibrium, $x_{ps}\left(t+1\right)=x_{ps}\left(t\right),x_{pr}\left(t+1\right)=x_{pr}\left(t\right)$, the iteration of the algorithm can be suspended and the optimal bandwidth resource allocation is obtained.

In each iteration, the two types of resource customers s and r calculate the prices they should pay to providers and the prices charged by downloading links according to the amount of resources provided by providers. Each provider p calculates the price charged by its upload link and updates the resource allocation for its customers. Therefore, we can repeat the iterative process until the optimum is finally reached.

## 6. Simulation and Numerical Examples in a Smart Home Environment

**Figure 3:**
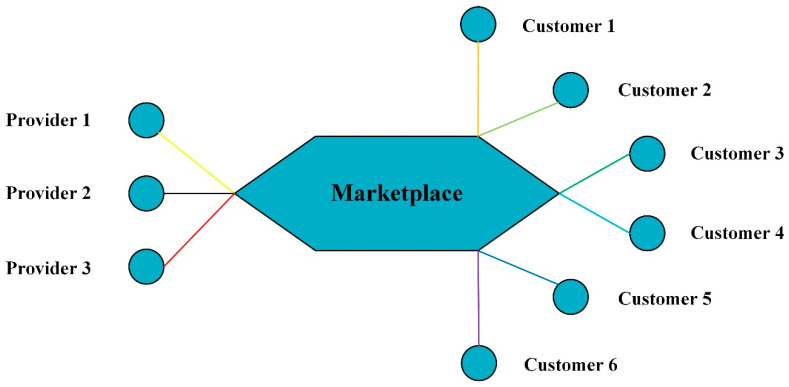
The simple topological structure of marketplace without using cloud center.

In this section, we will build a small-scale smart home environment in an edge computing resource allocation scenario without using cloud center and study the performance of resource allocation schemes. As shown in Figure 3, assume that there are 9 nodes (smart home applications) in the smart home environment, where 3 nodes on the left which serve as resource providers provide bandwidth resource for the other 6 nodes on the right which serve as customers. The resource providers and customers form a resource allocation market and are free to exchange resources in the marketplace.

### 6.1. Processing the Tasks for Customer s

Assume that the utility functions of 6 customers are: $U_{1}\left(y_{1}\right)=25\log\left(y_{1}+1\right),U_{2}\left(y_{2}\right)=20\log\left(y_{2}+1\right),U_{3}\left(y_{3}\right)=15\log\left(y_{3}+1\right),U_{4}\left(y_{4}\right)=10\log\left(y_{4}+1\right),U_{5}\left(y_{5}\right)=5\log\left(y_{5}+1\right),U_{6}\left(y_{6}\right)=4\log\left(y_{6}+1\right)$. Assume that the upstream capacity of providers for these customers is $\;C_{p}^{uL}=\left(C_{1}^{uL},C_{2}^{uL},C_{3}^{uL}\right)=\left(15,12,10\right)Mb/s$ and the downstream capacity of customers is $C_{s}^{d}=\left(C_{1}^{d},C_{2}^{d},C_{3}^{d},C_{4}^{d},C_{5}^{d},C_{6}^{d}\right)=\left(17,14,10,8,6,4\;\right)Mb/s$ and step sizes are $\kappa_{ps}=0.5$,$\;\alpha_{s}=0.2$,$\;\beta_{s}=0.2$,$\;\theta_{ps}=0.2$. The initial rates of the resource allocation are set to be $x_{ps}=1Mb/s$. 

**Figure 4:**
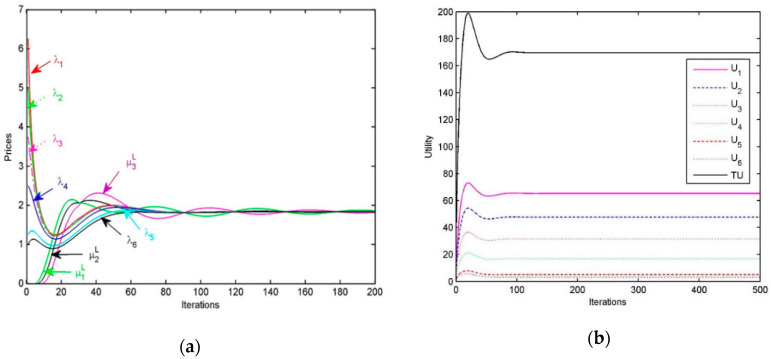
(**a**) Performance of the resource allocation algorithm for customer s: Prices; (**b**) Performance of the resource allocation algorithm for customer s: Utility.

The prices paid by the 6 resource customers are shown in Figure 4a, we find that the prices are finally driven to be an equilibrium, that is, $\lambda_{1}^{*}=\lambda_{2}^{*}=\lambda_{3}^{*}=\lambda_{4}^{*}=\lambda_{5}^{*}=\lambda_{6}^{*}$. The prices charged by the 3 providers for upload links are $\mu_{1}^{L},\mu_{2}^{L},\mu_{3}^{L}$, and at the optimum $\mu_{1}^{L*}=\;\mu_{2}^{L*}=\mu_{3}^{L*}$. After a certain number of iterations, the prices charged by providers for upload links and the prices paid by customers are equal, namely $\lambda_{s}^{*}=\mu_{p}^{L*},p\in P\left(s\right)$.

Figure 4b shows the utility of resource customers $U_{1},U_{2},U_{3},U_{4},U_{5},U_{6}$, as well as the aggregated utility $TU=U_{1}+U_{2}+U_{3}+U_{4}+U_{5}+U_{6}$. Obviously, the algorithm can drive the customer’s utility to reach the optimal value within a limited number of iterations. 

**Figure 5:**
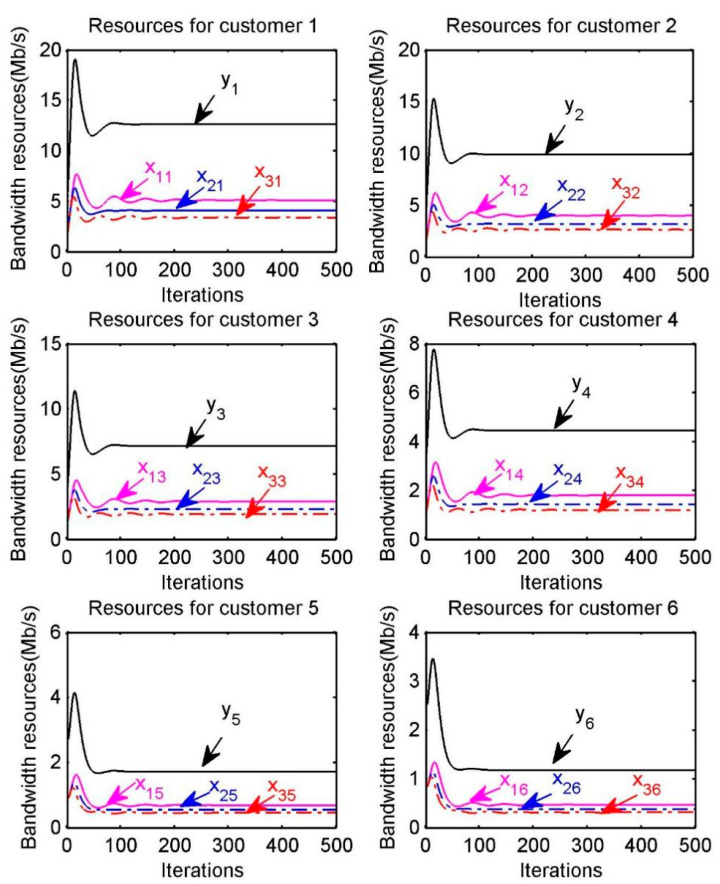
Optimal bandwidth resource allocation for customer s.

The optimal resource allocation for each customer is also shown in Figure 5, where $x_{ps}$ represents the amount of bandwidth resource provided by provider p to customer s, $y_{s}$ is the amount of bandwidth resource received by each customer s. For example, in the “Resources for customer 1” of Figure 5, $x_{11},x_{21},x_{31}$ respectively represent the amount of resource provided by providers p=1, p=2, p=3  to customer s=1 and $y_{1}$ represents the total amount of bandwidth resource obtained by the customer s=1. We can find that the optimal resource allocation can be achieved within a certain number of iterations. 

Linear Interactive and General Optimizer (LINGO) is a nonlinear programming software which can be used to solve nonlinear programming problems, some linear and nonlinear equations, and has become one of the best choices for solving optimization models with its powerful functions. As shown in Table 2, we list the simulation results of the proposed algorithm and the optimal solution of the optimization problem by applying LINGO. It is not difficult to find that the total amount of optimal bandwidth resource received by each resource customer is unique, but the optimal bandwidth resource obtained from each resource provider is not unique. This can be understood from the fact that a customer can obtain bandwidth resource from multiple providers and a provider can provide resource for multiple customers, which leads to the non-uniqueness of the optimal solution. Indeed, according to the convex optimization theory [52], the objective function is concave with respect to the variables and the constraints are linear, which means that the resource allocation model is a convex optimization problem. However, the objective function is not strict concave with respect to variables $x_{ps}$, thus the optimal bandwidth allocation from provider to each customer is not unique.

**Figure 6:**
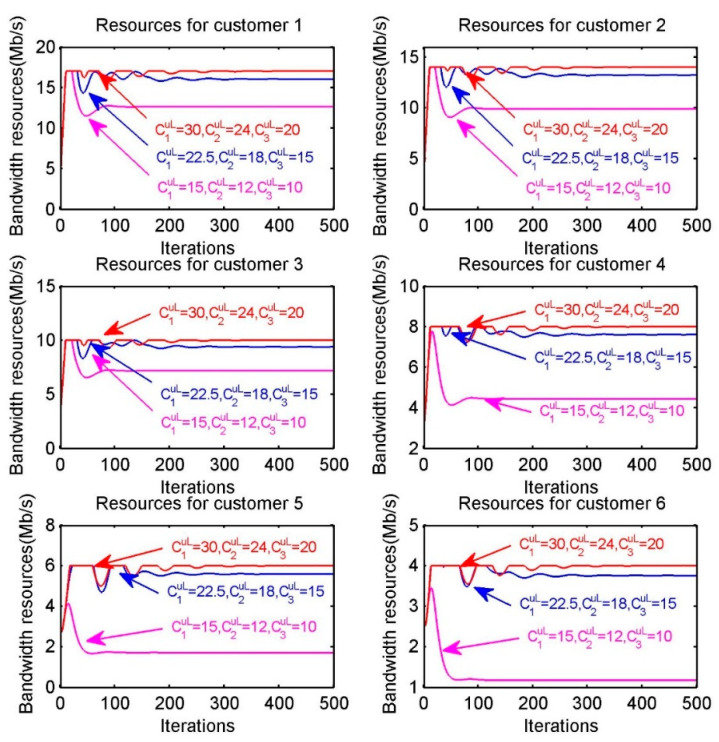
Optimal bandwidth resource allocation of each customer s when increasing the upload capacity of providers.

According to Equation (28), we can observe that optimal bandwidth resource allocation obtained by customer s not only relies on the willingness to pay (WTP) of customer, the number of customers in sub-region and the upload bandwidth capacity $C_{p}^{uL}$ of provider, but is also subject to the download bandwidth capacity $C_{s}^{d}$ of customer. In other words, when the amount of bandwidth resources provided by providers increases, a customer may not be able to pick up the entire amount of resources due to its own limited download bandwidth capacity. Therefore, we can determine whether a customer is able to pick up the entire amount of resources provided by providers through the assumption that the amount of resources provided by the providers increases, i.e., through releasing provider’s upload bandwidth capacity. As shown in Figure 6, we assume that the upload bandwidth capacity of providers in diverse settings are $\left(C_{1}^{uL},C_{2}^{uL},C_{3}^{uL}\right)=\left(15,12,10\right)Mb/s$, $\left(C_{1}^{uL},C_{2}^{uL},C_{3}^{uL}\right)=\left(22.5,18,15\right)Mb/s$, $\left(C_{1}^{uL},C_{2}^{uL},C_{3}^{uL}\right)=\left(30,24,20\right)Mb/s$, respectively. The six sub-figures correspond to the resource allocation for the six customers and in each sub-figure the red, blue and purple lines correspond to the resource allocation under different upload bandwidth capacities. Obviously, when the upload bandwidth capacity is released, the amount of resources received by customer is affected by its download bandwidth capacity.

**Figure 7:**
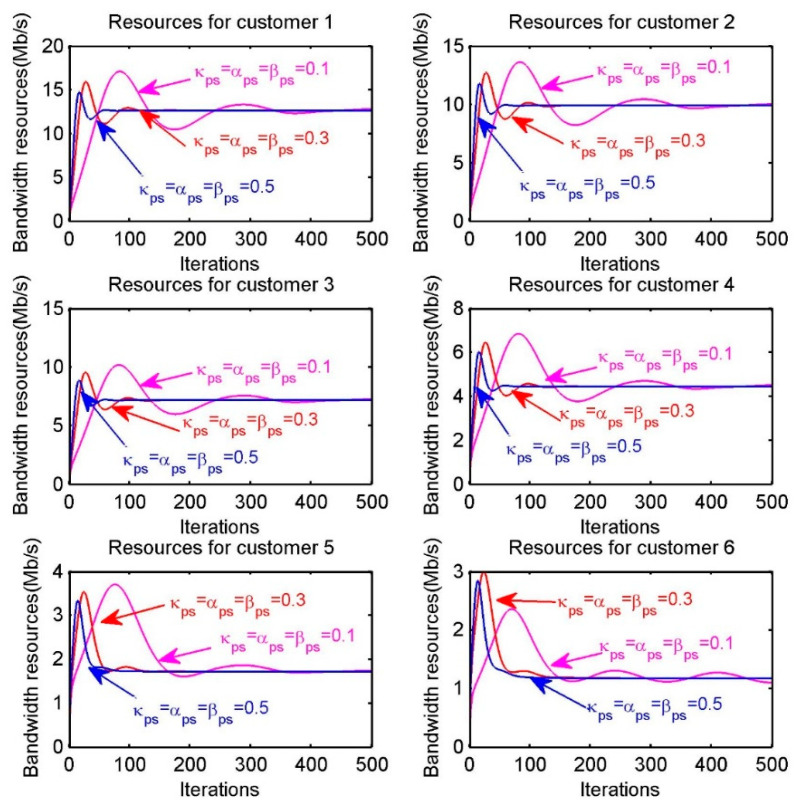
Resources allocation of each customer s when choosing different step sizes.

In Figure 7, we investigate the effect of different iterative step sizes on convergence speed. The six sub-figures correspond to the resource allocation for the six customers. The purple, red and blue lines in each sub-figure are regarded as resource allocation with different step sizes. We can generalize the effect of different iteration step sizes on convergence speed by comparing how fastly the lines of different colors finally converge in the sub-figure. For example, in the first sub-figure “Resources for customer 1”, the purple, red and blue lines represent the resource allocation in the case of $\kappa_{ps}=\alpha_{s}=\beta_{s}=0.1$, $\kappa_{ps}=\alpha_{s}=\beta_{s}=0.3$, $\kappa_{ps}=\alpha_{s}=\beta_{s}=0.5$, respectively. Obviously, the convergence speed is proportional to step sizes. Thus, it is essential that choosing appropriate step sizes for achieving the optimal resource allocation within reasonable iteration times.

### 6.2. Processing the Tasks for Customer r

Assume that the utility functions of customers for the tasks with high requirement on latency and low requirement on processing capacity are: $U_{1}\left(y_{1}\right)=100-\frac{50}{y_{1}},U_{2}\left(y_{2}\right)=100-\frac{40}{y_{2}},U_{3}\left(y_{3}\right)=100-\frac{30}{y_{3}},U_{4}\left(y_{4}\right)=50-\frac{50}{y_{4}},U_{5}\left(y_{5}\right)=50-\frac{40}{y_{5}},U_{6}\left(y_{6}\right)=50-\frac{30}{y_{6}}$. Assume that the upstream capacity of provider is $\;C_{p}^{uH}=\left(C_{1}^{uH},C_{2}^{uH},C_{3}^{uH}\right)=\left(10,8,5\right)Mb/s$, the downstream capacity of customer r is $C_{r}^{d}=\left(C_{1}^{d},C_{2}^{d},C_{3}^{d},C_{4}^{d},C_{5}^{d},C_{6}^{d}\right)=\left(20,18,15,12,10,5\right)Mb/s$ and the step sizes $\kappa_{pr}=0.5$, $\alpha_{r}=0.2$, $\beta_{r}=0.2$, $\;\theta_{pr}=0.2$. The initial rates of the resource allocation are all set to be $x_{pr}=0.5Mb/s$.

**Figure 8:**
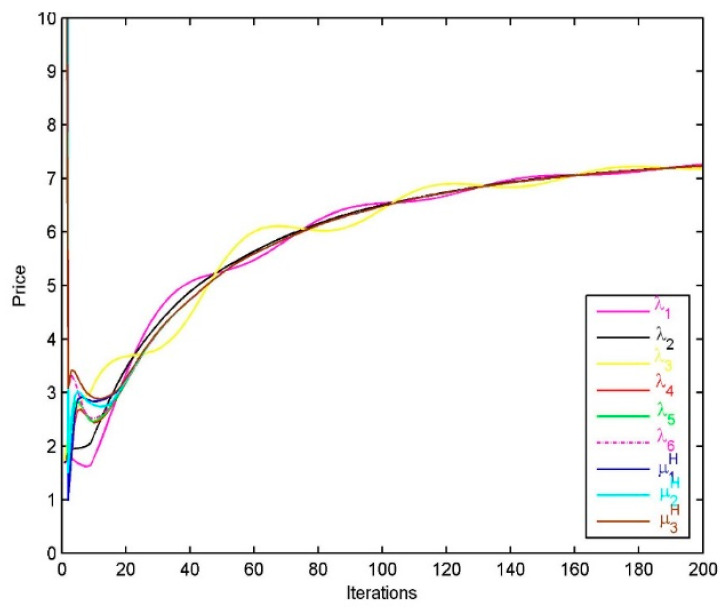
Performance of the resource allocation algorithm for customer r: Prices.

The prices charged by the three providers for upload links are shown in Figure 8. We find that at the equilibrium they are all equal. The prices paid by the six customers are also shown in this figure. It is obvious that at the equilibrium these prices are all equal, i.e.,$\;\lambda_{1}^{*}=\lambda_{2}^{*}=\lambda_{3}^{*}=\lambda_{4}^{*}=\lambda_{5}^{*}=\lambda_{6}^{*}$. Furthermore, after reasonable iterations, the prices charged by the providers and the prices paid by the resource customers are all equal, namely $\lambda_{r}^{*}=\mu_{p}^{H*},p\in P\left(r\right)$.

**Figure 9:**
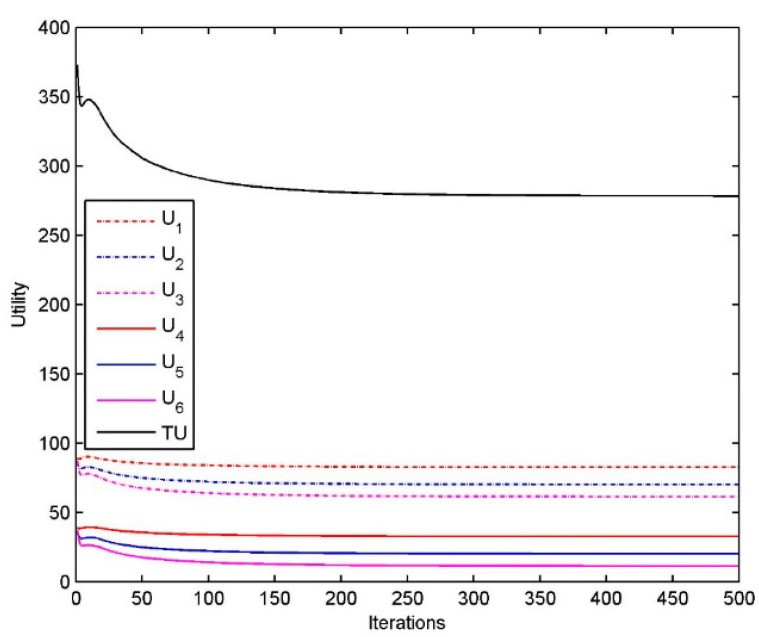
Performance of the resource allocation algorithm for customer r: Utility.

Figure 9 shows the utility of each resource customer $U_{1},U_{2},U_{3},U_{4},U_{5},U_{6}$, as well as the aggregated utility $TU=U_{1}+U_{2}+U_{3}+U_{4}+U_{5}+U_{6}$. Obviously, the algorithm can drive the customer’s utility to reach the optimal value within a limited number of iterations. The utility of each resource customer r is related to the number of tasks and time limits, from which we find that the longer the time limits are, the higher the utility of resource customer is and the larger the number of tasks for customer are, the lower the utility of customer is.

**Figure 10:**
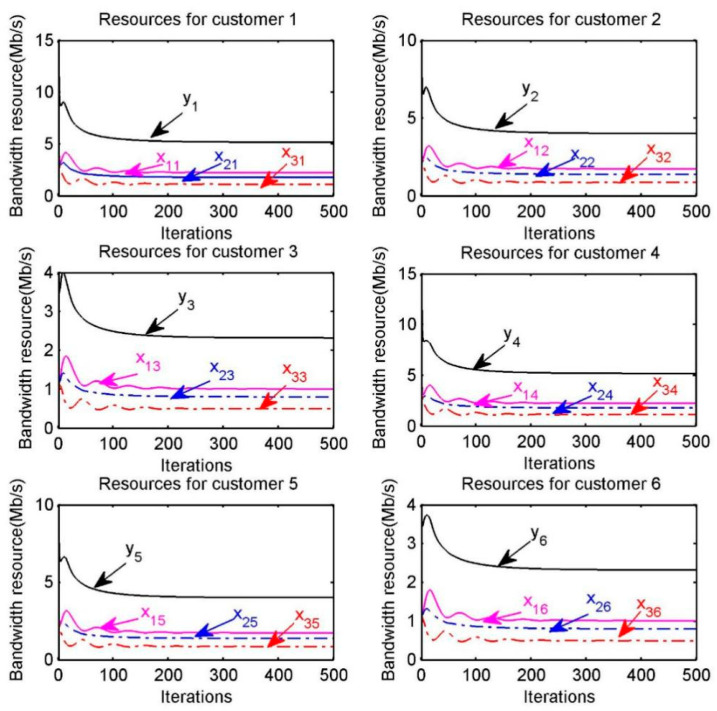
Optimal bandwidth resource allocation for customer r.

The optimal resource allocation is also shown in Figure 10, where $x_{pr}$ represents the amount of bandwidth resource provided by provider p to customer r, $y_{r}$ is the amount of bandwidth resource received by each customer r. Meanwhile, in the “Resource for customer 1” of Figure 10, $x_{11},x_{21},x_{31}$ respectively represents the amount of resource provided by the three providers to customer 1 and $y_{1}$ represents the total amount of resource obtained by customer 1. 

In Table 3, we list the optimal resource allocation obtained from the algorithm and the optimal value obtained by using the nonlinear programming software LINGO. It is not difficult to observe that the total amount of optimal bandwidth resources received by each resource customer is unique, which has been justified in Theorem 1 When the constraints with download links of customers are not active in the simple case, the optimal bandwidth allocation for each customer can also be derived from the algorithm, which is also equivalent to the optimal values from LINGO.

**Figure 11:**
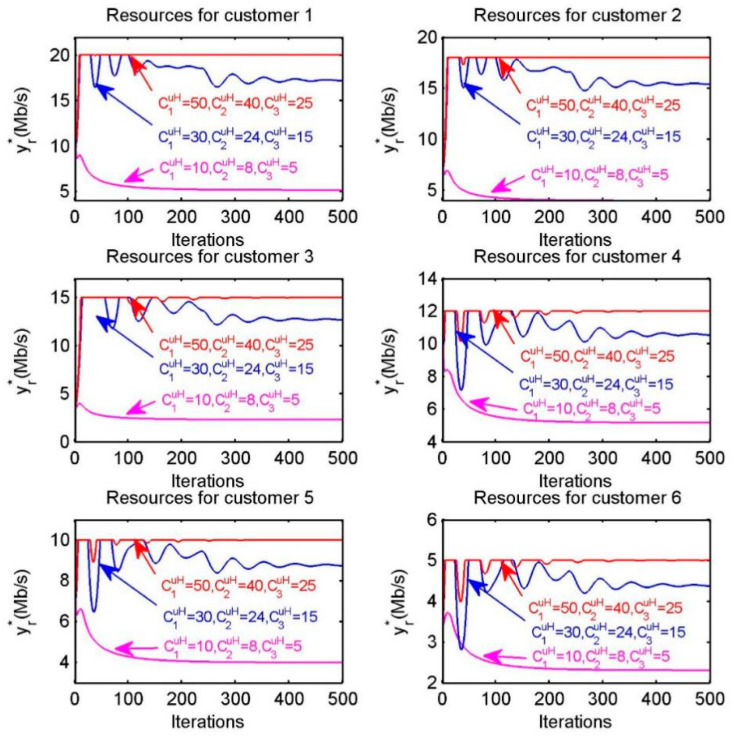
Resource allocation of each customer r when increasing the upload capacity of providers.

According to Equation (40), we can observe that optimal bandwidth resource allocation obtained by customer r not only depends on the amount of task $M_{r}$ of customer r and the upload bandwidth capacity $C_{p}^{uH}$ of provider p, but is also subject to the download bandwidth capacity $C_{r}^{d}$ of customer r. Therefore, we can discuss whether a customer is able to pick up the entire amount of resources provided by providers through the assumption that the amount of resources provided by the providers improves, i.e., increasing provider’s upload bandwidth capacity gradually. As shown in Figure 11, we assume that the upload bandwidth capacity of providers in diverse settings are $\left(C_{1}^{uH},C_{2}^{uH},C_{3}^{uH}\right)=\left(10,8,5\right)Mb/s$, $\left(C_{1}^{uH},C_{2}^{uH},C_{3}^{uH}\right)=\left(30,24,15\right)Mb/s$, $\left(C_{1}^{uH},C_{2}^{uH},C_{3}^{uH}\right)=\left(50,40,25\right)Mb/s$, respectively. 

In each sub-figure of the Figure 11, the red, blue and purple lines correspond to the resource allocation under different upload bandwidth capacities. Obviously, when the upload bandwidth capacity of each provider increases, the amount of resources received by each customer is constrained by its download bandwidth capacity. For instance, the red line in the sub-figure of “Resources for customer 1” is restricted within 20 Mb/s due to its download bandwidth capacity.

**Figure 12:**
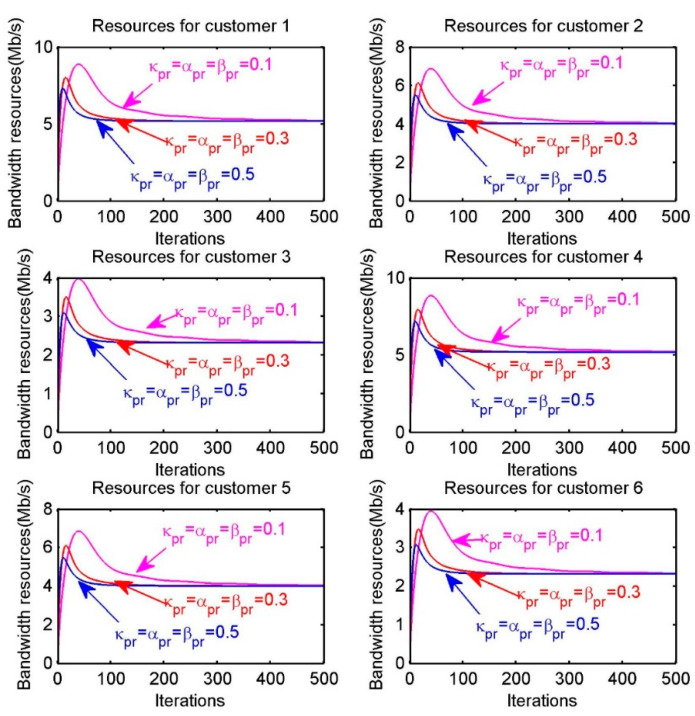
Resources allocation of each customer r when choosing different step sizes.

In Figure 12, we demonstrate the total bandwidth resource allocation of each customer, when different iteration step sizes are selected. For example, the first sub-figure “Resources for customer 1”, the blue curve with parameters $\kappa_{pr}=\alpha_{r}=\beta_{r}=0.5$ converges at a significantly faster speed than the purple curve with parameters $\kappa_{pr}=\alpha_{r}=\beta_{r}=0.1$. Based on the previous analysis and discussion on the convergence speed, we summarize that the convergence speed mainly depends on parameters such as step sizes rather than other factors. Therefore, we can get some conclusions, on the one hand, the iteration step sizes should be small enough to ensure convergence, however, it is unnecessary that step sizes are too small to slow the speed of convergence. On the other hand, the step sizes should also be not so large that the algorithm may not converge efficiently within the neighborhood of the optimum.

## 7. Conclusions

In recent years, the smart home market has developed rapidly and smart home products in various forms have begun to reach millions of households, greatly improving the convenience of people’s living. However, smart home applications are generally intelligent independently, which relies on the cloud platform to achieve remote control and realize collaborative work. Once attacked, there would be a serious threat and impact on the data privacy and security of smart homes. At the same time, as a decentralized computing framework, edge computing can provide networking, storage and computation services to IoT devices and reduce the bandwidth pressure of links. In a smart home environment, generally edge computing may face the issue of network latency and data security due to centralized cloud or data center, so edge computing without using cloud center has broad development potential as a new deployment choice for smart home. This paper discusses the bandwidth resource allocation of edge computing without using cloud center for processing tasks which are decomposed into two types, the tasks with low requirement on latency and high requirement on processing capacity, and the tasks with high requirement on latency and low requirement on processing capacity in a smart home environment. According to the characteristics of different types of tasks, the utility maximization model of edge computing resource allocation is established and further interpreted from an economic point of view. We analyze the relationships between the prices charged by providers (fog nodes) for upload links and the prices paid by customers (users), and propose a gradient-based algorithm which can achieve optimal resource allocation for different tasks. Finally, some numerical examples are given to illustrate the effectiveness and convergence of the proposed algorithm.

## Figures and Tables

**Table 1:** Notation list.

Notations	Meanings
S	the set of resource customers (users) for the tasks with low requirement on latency and high requirement on processing capacity, each element is customer s
R	the set of resource customers (users) for the tasks with high requirement on latency and low requirement on processing capacity, each element is customer r
P	the set of resource providers (edge nodes), each element is provider p
P(s)	the set of providers offering resources for customers s∈S
P(r)	the set of providers offering resources for customers r∈R
S(p)	the set of customers S that request resource from provider p
R(p)	the set of customers R that request resource from provider p
$x_{ps}$	the resources granted by provider p to customer s
$x_{pr}$	the resources granted by provider p to customer r
$y_{s}$	the aggregate resources granted by providers to customer s $y_{s}=\sum_{p:p\in P\left(s\right)}x_{ps}$
$y_{r}$	the aggregate resources granted by providers to customer r $y_{r}=\sum_{p:p\in P\left(r\right)}x_{pr}$
$z_{p}^{L}$	the resources provided by provider p to all its customers, $z_{p}^{L}=\sum_{s:s\in S\left(p\right)}x_{ps}$
$z_{p}^{H}$	the resources provided by provider p to all its customers, $z_{p}^{H}=\sum_{r:r\in R\left(p\right)}x_{pr}$
$U_{s}\left(y_{s}\right)$	the utility generated by the customer s
$U_{r}\left(y_{r}\right)$	the utility generated by the customer r
$C_{s}^{d}$	the capacity of bandwidth resource of the downstream links of customers s
$C_{r}^{d}$	the capacity of bandwidth resource of the downstream links of customers r
$C_{p}^{uL}$	the capacity of reserved bandwidth resource of the upstream link of provider p for customers s
$C_{p}^{uH}$	the capacity of reserved bandwidth resource of the upstream link of provider p for customer r
$\omega_{s}$	the willingness to pay (WTP) of customer s for processing the tasks
$M_{r}$	the amount of task for customer r
$T_{r}$	the time limit for customer r
$B_{r}$	the minimum bandwidth requirement for customer r during resource allocation

**Table 2:** The optimal bandwidth allocation for customer s.

**Variable**	$x_{11}^{*}$	$x_{21}^{*}$	$x_{31}^{*}$	$x_{12}^{*}$	$x_{22}^{*}$	$x_{32}^{*}$	$y_{1}^{*}$	$y_{2}^{*}$
Algorithm	5.116	4.088	3.404	4.015	3.205	2.666	12.60	9.886
LINGO	4.482	3.396	4.729	4.726	3.190	1.969	12.60	9.885
**Variable**	$x_{13}^{*}$	$x_{23}^{*}$	$x_{33}^{*}$	$x_{14}^{*}$	$x_{24}^{*}$	$x_{34}^{*}$	$y_{3}^{*}$	$y_{4}^{*}$
Algorithm	2.901	2.324	1.940	1.804	1.441	1.199	7.165	4.444
LINGO	3.121	2.178	1.865	1.512	1.621	1.309	7.164	4.442
**Variable**	$x_{15}^{*}$	$x_{25}^{*}$	$x_{35}^{*}$	$x_{16}^{*}$	$x_{26}^{*}$	$x_{36}^{*}$	$y_{5}^{*}$	$y_{6}^{*}$
Algorithm	0.696	0.559	0.467	0.475	0.382	0.320	1.722	1.177
LINGO	1.157	0.438	0.127	0	0.177	0	1.722	1.177

**Table 3:** The optimal allocation of the bandwidth resources for customer *r.*

**Variable**	$x_{11}^{*}$	$x_{21}^{*}$	$x_{31}^{*}$	$x_{12}^{*}$	$x_{22}^{*}$	$x_{32}^{*}$	$y_{1}^{*}$	$y_{2}^{*}$
Algorithm	2.244	1.800	1.133	1.744	1.395	0.871	5.177	4.010
LINGO	3.650	0.811	0.714	1.480	1.511	1.019	5.175	4.010
**Variable**	$x_{13}^{*}$	$x_{23}^{*}$	$x_{33}^{*}$	$x_{14}^{*}$	$x_{24}^{*}$	$x_{34}^{*}$	$y_{3}^{*}$	$y_{4}^{*}$
Algorithm	1.010	0.806	0.500	2.245	1.800	1.134	2.316	5.179
LINGO	0.904	0.967	0.444	1.858	1.921	1.397	2.315	5.176
**Variable**	$x_{15}^{*}$	$x_{25}^{*}$	$x_{35}^{*}$	$x_{16}^{*}$	$x_{26}^{*}$	$x_{36}^{*}$	$y_{5}^{*}$	$y_{6}^{*}$
Algorithm	1.746	1.395	0.869	1.011	0.806	0.498	4.010	2.315
LINGO	1.309	1.372	1.328	0.800	1.418	0.098	4.009	2.316
